# Surface Modification Strategies for Chrysin-Loaded Iron Oxide Nanoparticles to Boost Their Anti-Tumor Efficacy in Human Colon Carcinoma Cells

**DOI:** 10.3390/jfb15020043

**Published:** 2024-02-13

**Authors:** Aynura Karimova, Sabina Hajizada, Habiba Shirinova, Sevinj Nuriyeva, Lala Gahramanli, Mohammed M. Yusuf, Stefano Bellucci, Christoph Reissfelder, Vugar Yagublu

**Affiliations:** 1 Nanoresearch Laboratory, Baku State University, Baku AZ 1148, Azerbaijan; aynurakarimova@bsu.edu.az (A.K.); habibashirinova@bsu.edu.az (H.S.); sevinc.nuriyeva@bsu.edu.az (S.N.); lalagahramanli@bsu.edu.az (L.G.); 2 Department of Surgery, Medical Faculty Mannheim, University of Heidelberg, 68167 Mannheim, Germany; sabina.hajizada@medma.uni-heidelberg.de (S.H.); mohammedmohiuddin.yusuf@medma.uni-heidelberg.de (M.M.Y.); christoph.reissfelder@umm.de (C.R.); 3 Laboratori Nazionali di Frascati, Istituto Nazionale di Fisica Nucleare, 00044 Frascati, Italy; stefano.bellucci@lnf.infn.it

**Keywords:** magnetite nanoparticles, coating agents, loading efficiency, drug concentration, colon cancer

## Abstract

Enhancing nanoparticles’ anti-cancer capabilities as drug carriers requires the careful adjustment of formulation parameters, including loading efficiency, drug/carrier ratio, and synthesis method. Small adjustments to these parameters can significantly influence the drug-loading efficiency of nanoparticles. Our study explored how chitosan and polyethylene glycol (PEG) coatings affect the structural properties, drug-loading efficiency, and anti-cancer efficacy of Fe_3_O_4_ nanoparticles (NPs). The loading efficiency of the NPs was determined using FTIR spectrometry and XRD. The quantity of chrysin incorporated into the coated NPs was examined using UV–Vis spectrometry. The effect of the NPs on cell viability and apoptosis was determined by employing the HCT 116 human colon carcinoma cell line. We showed that a two-fold increase in drug concentration did not impact the loading efficiency of Fe_3_O_4_ NPs coated with PEG. However, there was a 33 Å difference in the crystallite sizes obtained from chitosan-coated Fe_3_O_4_ NPs and drug concentrations of 1:0.5 and 1:2, resulting in decreased system stability. In conclusion, PEG coating exhibited a higher loading efficiency of Fe_3_O_4_ NPs compared to chitosan, resulting in enhanced anti-tumor effects. Furthermore, variations in the loaded amount of chrysin did not impact the crystallinity of PEG-coated NPs, emphasizing the stability and regularity of the system.

## 1. Introduction

Resistance to chemotherapy is a major challenge in the effective treatment of various cancers, including colorectal cancers. Nanoparticles (NPs) are gaining attention as a powerful tool for preventing chemoresistance due to their improved stability, biocompatibility, enhanced permeability, and precise targeting [1]. The advantages of NPs include sustained drug release over time, the maintenance of therapeutic concentrations in the tumor, and the potential overcoming of resistance associated with the rapid excretion of free drugs [2]. Novel functionalized magnetic NPs play a critical role in the delivery of various drugs and drug candidates, including promising therapeutic biomacromolecules, specific cell types, individual cells, or specific intracellular compartments, such as the nucleus and mitochondria [3]. Currently, nanosized iron oxide particles are widely employed in oncological studies as drug carriers. This is due to their low toxicity, stability in aqueous solutions, biocompatibility, and ability to facilitate the magnetic hyperthermia of tumor cells [4,5,6].

However, the success rate that leads to nanoparticles being used on a large scale as clinical products is low [7]. The first cancer nanomedicine, Doxil^®^, was the first FDA-approved liposomal nanoparticle formulation for the treatment of certain cancers [8]. Iron oxide nanoparticles, such as Feridex, Combidex, Feraheme, and NanoTherm are some of the FDA-approved agents for both cancer imaging and therapy applications [9]. Despite the advantages of iron oxide nanoparticles for drug delivery and diagnostics, numerous animal experiments have confirmed their toxicity to organs, such as the nervous system, heart, lungs, thyroid, liver, and lymph nodes [10,11]. Therefore, it became an important task to develop strategies to prevent the opsonization of nanoparticles to extend their circulation, enhance their carrier capacity, and improve targeted delivery. A recently reported innovative approach using magnetic biohybrid microrobot multimers (BMMs) based on chlorella demonstrated significant promise for targeted drug delivery [12]. Reduced toxicity may be achieved by improving iron oxide nanoparticles’ carrier capacity and introducing them in smaller, more potent doses.

The major disadvantage of unmodified NPs is their nonspecific interactions with cells, leading to accumulation outside target organs [13]. Moreover, unmodified NPs have high toxicity and low colloidal stability in physiological media [14]. The careful surface modification of NPs, i.e., functionalization with biocompatible agents, can mitigate these challenges and enable the maintenance of both magnetic characteristics and the optimization of drug carrier capability [15,16,17]. Colloid modification can be achieved through the use of monomeric stabilizers (such as carboxylates, phosphates, and sulfates) or polymeric stabilizers (such as polyacrylic acid, polyethyleneimine, and polyethylene glycol) [18,19,20,21,22,23]. Polymers are widely used as a shell covering the surfaces of nanomaterials. They can prevent NP oxidation and provide nanoparticles with collateral stability [24]. Moreover, the polymer coating can improve NP biocompatibility and reduce their toxicity [25]. In addition, coating with polymers can be arranged to respond to specific triggers, such as changes in temperature, pH, NPs, and polymer concentration ratios, allowing for substance-controlled release [26].

Selecting the coating agent and method for NPs is critical to ensure their efficiency as drug carriers [27,28]. It is imperative that the chosen coating preserves the loading capacity of the NPs, facilitating the effective encapsulation of therapeutic agents while maintaining the stability of the drug carrier [13]. The coating method is the most frequently employed surface modification approach to conjugate organic and inorganic materials onto the surface of magnetic NPs [22]. In this case, modification can be performed according to the encapsulation strategy or through the formation of a self-assembled monolayer onto the particles’ surfaces [29]. Coating with polymers is widely used to modify the surface of NPs to prevent aggregation and produce stable suspensions [30]. Our current study focuses on the synthesis and characterization of iron oxide NPs modified by coating their surfaces with various polymer materials at different concentrations. These NPs were further loaded with the natural anti-cancer bioflavonoid chrysin to achieve increased anticancer efficacy. Chrysin, also known as 5,7-dihydroxyflavone or 5,7-dihydroxy-2-phenyl-4H-chromen-4-one, has demonstrated significant anti-carcinogenic, anti-inflammatory, and anti-oxidant properties [31]. Magnetite NPs (Fe_3_O_4_, diameter ~20–25 nm) were chosen as a nucleus due to their previously demonstrated low toxicity and biodegradability. PEG and chitosan were employed for designing the polymeric shell of the NPs because of their biocompatibility and non-toxic characteristics. The anti-cancer efficacy of the NPs was studied using HCT-116 human colon cancer cells in in vitro conditions.

## 2. Materials and Methods

### 2.1. Materials

Iron (III) chloride tetrahydrate (FeCl_2_·4H_2_O), iron (III) chloride hexahydrate (FeCl_3_·6H_2_O), polyethylene glycol (PEG, MW6000), chitosan (degree of deacetylation <90%, MW177.20), ammonium hydroxide (NH_4_OH, 23–25%), acetic acid (CH_3_COOH, 99%), chrysin (C_15_H_10_O_4_, 97%), and N, N-dimethylformamide (HCON(CH_3_)_2_, ≥99%) were purchased from KarmaLab (Izmir, Turkey). All of the used chemicals were of analytical grade.

### 2.2. Methods

#### 2.2.1. Preparation of PEG and Chitosan-Coated Nanoparticles

The synthesis of the magnetite NPs involved the chemical co-precipitation of ferric and ferrous salts under the presence of nitrogen gas [32]. FeCl_3_·6H_2_O and FeCl_2_·4H_2_O with a 2:1 mole ratio were dissolved in 100 mL of deionized water. The solution was heated under vigorous stirring until 70 °C was reached for 30 min. For the PEG coating, an appropriate amount of polymer was slowly added to the solution. After stirring for 60 min, chemical precipitation was achieved at a stirring rate of 400 rpm by adding 10–15 mL of NH_4_OH (23–25%) drop-wise until the pH reached 10, and then the temperature was raised to 90 °C. After the system was cooled to room temperature, the precipitate was separated with a NdFeB magnet and washed with deionized water and ethanol in the ratio of 2:1, respectively, several times until the pH was neutral. This step was required for the NPs’ further characterization. Finally, the PEG-coated magnetite NPs were dried in an oven at 80 °C for 4 h. Coating with chitosan was implemented by first preparing the chitosan solution. For this, 0.25 g of fine chitosan powder was dissolved in a mixture of 25 mL of deionized water and 1.25 mL of acetic acid. The chitosan-coated magnetite NPs were obtained using a previously described method [33].

The loading of chrysin into the PEG-magnetite NP system was performed using the adsorption method. Briefly, a solution of the anti-cancer drug dissolved in N, N-dimethylformamide, was added to the PEG-magnetic iron oxide NP powder, and the mixture was stirred with a mechanical stirrer at 700 rpm for 6 h at 25 °C. At the end of the procedure, free drug molecules were removed via magnetic cleaning. The resulting samples, PEG-magnetite NPs-chrysin, were then washed three times and dried in an oven at 40 °C for 4 h. The chrysin-loaded chitosan-stabilized magnetite NPs were synthesized using the same protocol.

In this research, the influence of drug concentration and coating agent type on drug-loading efficiency was investigated. To achieve this, the chrysin concentration was varied between 250 mg, 500 mg, and 1000 mg (Table 1).

#### 2.2.2. Characterization of Coated and Drug-Loaded Magnetite Nanoparticles

The structure and drug-loading efficiency of the iron oxide NPs coated with various polymers were investigated using X-ray diffraction (XRD), Fourier-transform infrared (FTIR) spectroscopy, and visible ultraviolet (UV–Vis) spectroscopy. To evaluate the crystalline nature and size characterization of the samples, XRD analysis was conducted using a Rigaku Mini Flex 600 X-ray diffractometer with high-intensity Cu–Kα radiation and 2*θ* ranging from 10° to 80°. Regarding the structure of the magnetite NPs with various coating agents, a FTIR spectrometer Varian 3600 with KBr pellets was used. In this case, the spectrum was observed from 4000–4500 cm^−1^. Additionally, the drug-loading efficiency for all the samples was investigated using a Specord 250 PLUS UV/Vis spectrometer.

#### 2.2.3. Calculation of Drug-Loading Efficiency (LE)

The suspensions of the drug-loaded Fe_3_O_4_ polymer-coated samples were centrifuged at 15,000 rpm for 20 min. The loading efficiency (LE) for the samples was determined by quantifying the absorption of the clear supernatant using UV–Vis spectroscopy. The absorbance values of chrysin were measured on a UV–Vis spectrum at a wavelength of 276.4 nm. The percentage of the LE of chrysin in the PEG and chitosan-coated magnetite NPs was determined using the following equation, respectively, as reported earlier [20]:

$$LE=\frac{\left(D_{t}-D_{f}\right)}{D_{t}}\times 100\%$$

where *D_t_* is the total amount of chrysin and *D_f_* is the amount of free chrysin in the supernatant after centrifugation.

#### 2.2.4. Cell Culture and MTT Assay

The anti-cancer efficacy of selected Fe_3_O_4_ NPs was studied using HCT 116 human colon cancer cells. HCT 116 cells were maintained in DMEM medium containing 10% FBS and 1% PSN under a humidified atmosphere (5% CO_2_) at 37 °C. Once 75–80% confluence was achieved, the cells were re-equilibrated with trypsin (0.25%) and EDTA (0.52 mM) in PBS and harvested at the appropriate density for 24 h before the cell viability assessment. In this assay, the HCT 116 cells were treated with chrysin-loaded Fe_3_O_4_ NPs coated with both PEG and chitosan at different concentrations (0–40 µg/mL) for 24 h. After treatment, the cells were rinsed with PBS, and MTT solution was added to form formazan salt. Subsequently, the media were replaced with DMSO (200 mL) to dissolve the formed formazan crystals in each well. The viability of the cells was spectrophotometrically determined at 570 nm using an ELISA reader (Emax, Molecular Devices, San Jose, CA, USA) to identify the number of viable cells, and the percentage of viability was calculated using the following equation: (1 − [ODt/ODc]) × 100%, where ODt is the mean optical density of wells treated with the tested sample and ODc is the mean optical density of untreated cells. All the treatment experiments were performed in triplicates.

#### 2.2.5. Apoptosis Assay

Early and late apoptotic cells were analyzed using the Annexin V-FITC Apoptosis Detection Kit I (BD Biosciences Pharmingen, Franklin Lakes, NJ, USA), following the recommended instructions. The cells were seeded in a 25 cm^2^ tissue culture flask (1 × 10^6^ cells). After overnight incubation, the cells were exposed to 5 μg/mL of Fe_3_O_4_@PEG500 and Fe_3_O_4_@Chtiosan500 in a 1:1 PEG-chrysin and chitosan-chrysin ratio. A positive control was included consisting of 5 μg/mL of chrysin, a concentration previously validated for its efficacy against HCT 116 cells [34,35]. The cells were trypsinized and rinsed twice with phosphate-buffered saline-1% bovine serum albumin-ethylenediaminetetraacetic acid, then resuspended in 100 μL of 1× binding buffer. Subsequently, an aliquot of 2.5 μL of propidium iodide (PI) and 0.5 μL of fluorescein isothiocyanate (FITC) were added, and this was allowed to react for 15 min. Lastly, the fluorescence of the cells was measured after adding 100 μL of 1× binding buffer before being evaluated using BD FACSCanto™ II flow cytometers (BD Biosciences, New York, NY, USA, by Maxwell Becton and Fairleigh S. Dickinson) with FlowJo™ Software version 10.10 (Ashland, OR, USA: Becton, Dickinson & Company, 2017).

#### 2.2.6. Statistical Analysis

All statistical analyses were conducted using JMP software version 16 (SAS Institute Inc., Cary, NC, USA). The differences among multiple groups were analyzed using a one-way ANOVA, followed by Tukey’s method for post-hoc comparisons. Differences with *p* < 0.05 were considered statistically significant.

#### 2.2.7. Declaration of Generative AI in Writing This Manuscript

We declare that the manuscript creation process did not involve the use of any AI tools to develop ideas, generate text, or improve the overall quality of content. During the writing process, conventional writing and proofreading tools such as Grammarly were involved to ensure grammatical accuracy and language refinement.

## 3. Results

### 3.1. Characterization and Structural Properties

**Figure 1:**
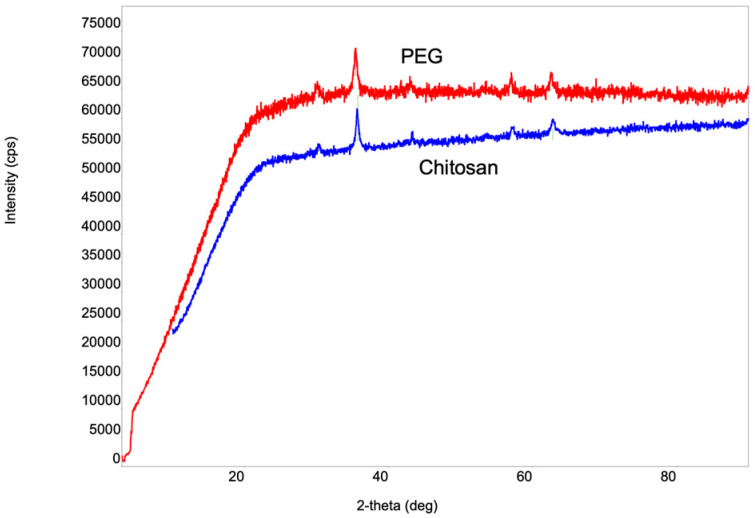
X-ray diffractogram of Fe_3_O_4_ NPs coated with PEG and chitosan. The crystal structure of the magnetite (Fe_3_O_4_) NPs synthesized via chemical co-precipitation and coated with PEG and chitosan was analyzed. Characteristic X-ray lines of magnetite NPs were observed in both samples. Therefore, the d-spacing values of the significant peaks match well (ICDD DB card number 01-073-9877).

Examining the phase arrangement of the synthesized NPs holds significance in establishing correlations with various physicochemical properties. For phase identification, we employed XRD analysis. The selection of this method was based on its simplicity and its non-destructive impact on the samples. Figure 1 shows the X-ray structural images of the Fe_3_O_4_ NPs coated with various stabilizers.

The values of the 2-theta angle and crystallite size for the iron oxide NPs coated with PEG and chitosan are presented in Table 2. The values of the 2-theta angles obtained for both cases correspond to spinal-structured Fe_3_O_4_ particles with a cubic crystal lattice. Table 2 shows that the values of the 2-theta angles for the chitosan-coated nanoparticles shift to relatively larger angles compared to PEG-coated Fe_3_O_4_ nanoparticles, which is manifested by the relative decrease in nanoparticle size.

**Figure 2:**
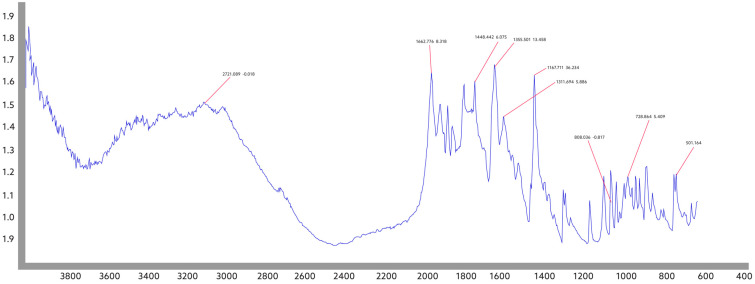
FTIR spectrum of pure chrysin. FTIR spectrum for pure chrysin allows the evaluation of its molecular state.

FTIR spectral analysis was conducted to determine the presence of functional groups in the samples. In addition to the synthesized samples, an FTIR spectrum was recorded for pure chrysin (Figure 2).

The absorption band at 3012 cm^−1^ observed in the FTIR spectrum of chrysin corresponds to OH groups, the absorption band at 2926 cm^−1^ corresponds to CH groups, and the absorption band at 1652 cm^−1^ corresponds to the C=O carbonyl group. Additionally, the bands at 1610 cm^−1^, 1355 cm^−1^, 1245 cm^−1^, and 1167 cm^−1^ demonstrate the chain vibrations of C-C, C-OH, C=C, and C-O-C groups.

**Figure 3:**
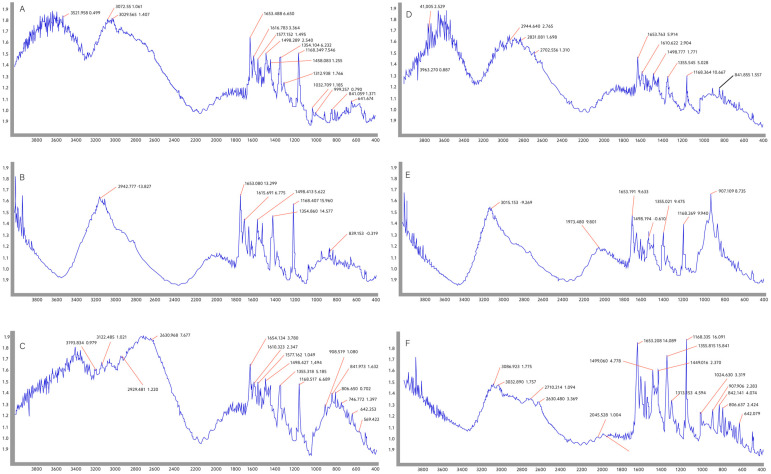
FTIR spectra of the samples obtained after attaching chrysin on Fe_3_O_4_@PEG and Fe_3_O_4_@chitosan in different ratios: (**A**) Fe_3_O_4_@PEG250 with a 1:0.5 PEG-chrysin ratio; (**B**) Fe_3_O_4_@PEG500 with a 1:1 PEG-chrysin ratio; (**C**) Fe_3_O_4_@PEG1000 with a 1:2 PEG-chrysin ratio; (**D**) Fe_3_O_4_@Chitosan250 with a 1:0.5 chitosan-chrysin ratio; (**E**) Fe_3_O_4_@Chitosan500 with a 1:1 chitosan-chrysin ratio; (**F**) Fe_3_O_4_@Chitosan1000 with a 1:2 chitosan-chrysin ratio. The characteristic peaks in the spectra indicate that PEG and chitosan-coated magnetite NPs were successfully synthesized, and the chrysin drug was loaded into them.

The FTIR spectrum of the samples obtained after attaching different amounts of chrysin on PEG and chitosan-coated magnetite NPs was comparatively studied (Figure 3A,B).

All chrysin-specific absorption bands were observed in the chrysin-loaded Fe_3_O_4_@PEG/samples (Figure 3A–C). The absorption bands at 1633 cm^−1^ and 1724 cm^−1^ observed in all the samples belong to PEG [35], and the changes observed in the range of 400–600 cm^−1^ belong to the Fe_3_O_4_ NPs [36]. There was no significant difference in the FTIR spectra of the chrysin Fe_3_O_4_@PEG system when varying the amount of the drug. For these samples, a slight change in the loading efficiency was determined depending on the initial amount of the drug (Table 3), which was confirmed by the almost unchanged structure in the FTIR spectra.

In the FTIR spectrum of the chrysin-loaded Fe_3_O_4_@Chitosan system (Figure 3D–F), all absorption bands belonging to chrysin were observed. In the spectrum, the bands corresponding to 2921 cm^−1^ and 2877 cm^−1^ correspond to chitosan, and the peaks in the range of 400–600 cm^−1^ coincide with the absorption bands of Fe_3_O_4_. The intensity of the chitosan absorption bands, which is clearly visible in the spectrum at a 1:0.5 concentration of Fe_3_O_4_ and the drug substance, is weakened at the 1:1 and 1:2 concentrations. This shows that the amount of chrysin in the system increased (Table 3).

**Figure 4:**
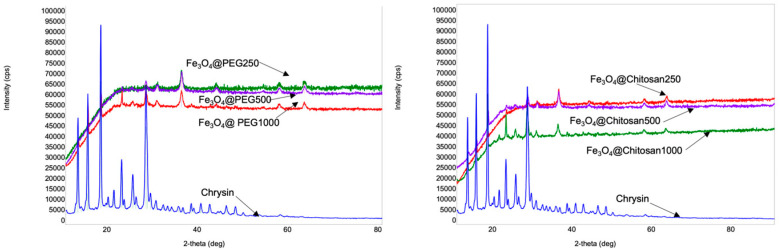
X-ray diffractograms of the obtained samples based on Fe_3_O_4_ nanoparticles coated with different polymers and loaded with various amounts of chrysin and pure chrysin. Fe_3_O_4_@PEG250, Fe_3_O_4_@PEG500 and Fe_3_O_4_@PEG1000 accordingly correspond to the Fe_3_O_4_@PEG and chrysin ratios of 1:0.5, 1:1, and 1:2. Fe_3_O_4_@Chtiosan250, Fe_3_O_4_@Chitosan500, and Fe_3_O_4_@Chitosan1000 accordingly correspond to Fe_3_O_4_@Chitosan and chrysin ratios of 1:0.5, 1:1, and 1:2.

The comparative studies of the X-ray diffractograms were conducted on the samples obtained from magnetite NPs with different coating agents and loaded with various amounts of chrysin (Figure 4).

The results obtained from the UV–Vis and XRD analyses are presented in a comparative manner in Table 3. The average crystallite sizes of the magnetite NPs with different coatings were calculated using Scherrer’s equation according to the diffraction peak at 35.4° [37]. Chrysin was detected at 276.4 nm. The drug-loading efficiency was determined as follows: LE = (The total amount of chrysin-the amount of free chrysin/the total amount of chrysin) × 100%.

A decrease in the size of the crystallites was observed in the chitosan-coated iron oxide NPs, with an increase in the amount of loaded chrysin in the NPs. In contrast, for PEG-stabilized NPs, no such regularity was observed between the drug concentration and crystallite size. There was a slight difference between the drug-loading efficiency values in Fe_3_O_4_@PEG500 and Fe_3_O_4_@PEG1000. There was a two-fold increase in the amount of drug compared to the amount of magnetite NPs, which did not lead to a significant increase in the loading efficiency. When chitosan was used as a coating material, the difference between the chrysin loading efficiency (Fe_3_O_4_@Chitosan500 and Fe_3_O_4_@Chitosan1000) values increased to 8%. While the variation of the drug amount depending on the amount of PEG-coated magnetite NPs had no significant effect on the loading efficiency, this was not observed in the case of chitosan-coated magnetite NPs. At the same time, when we look at the X-ray spectrum of pure chrysin, we see intense X-ray lines at 12.7°, 15°, 17.8°, and 27.8° values of 2-theta angles, which shows that chrysin has a highly crystalline nature. After loading chrysin on the surface of the nanoparticles, although the position of the characteristic peaks of chrysin was retained for all samples, their intensity decreased dramatically, and some of them completely disappeared. In the X-ray spectra of the samples coated with chitosan, although the intensities of the characteristic chrysin lines were reduced, they can be observed (Fe_3_O_4_@Chitosan250, Fe_3_O_4_@Chitosan500, and Fe_3_O_4_@Chitosan1000). In contrast, for the samples coated with PEG on the surface, only characteristic curve lines were partially observed for the Fe_3_O_4_@PEG1000 sample, where the amount of drug was twice the amount of nanoparticle. For the other samples (Fe_3_O_4_@PEG250 and Fe_3_O_4_@PEG500), most of the characteristic chrysin lines were overlapped by polymer noise. The available data suggest that this change in the X-ray spectrum of a drug with a high degree of crystallinity confirms its coverage on the nanoparticle surface [38,39,40]. Our X-ray studies show that drug-loading was more effective in PEG-coated nanoparticles. These results are in agreement with the results obtained from UV–Vis spectral analysis.

### 3.2. In-Vitro Cytotoxicity Analysis and Apoptosis Assay

**Figure 5:**
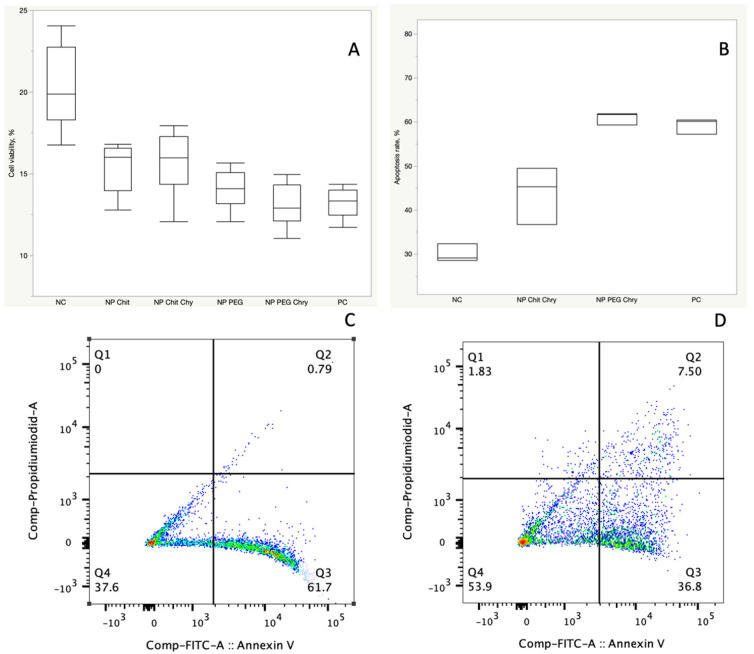
Cell viability and apoptosis rate under treatment with Fe_3_O_4_ NPs coated with PEG and chitosan. (**A**) Cell viability assessment using the MTT test with a 5 µg/mL treatment of Fe_3_O_4_@PEG500 with a 1:1 PEG-chrysin ratio and with 5µg/mL of Fe_3_O_4_@Chitosan500 with a 1:1 chitosan-chrysin ratio (P_NP_PEG_Chry_ vs. _NP_Chit_Chry_ = 0.0033). NC = negative control, where no treatment was administered. PC = positive control, where a treatment with 5 μg/mL of pure chrysin was applied. (**B**) Apoptosis rates following 48 h of treatment with Fe_3_O_4_@PEG500 and Fe_3_O_4_@Chitosan500 NPs, comparing the effects of coating with PEG versus chitosan. NC = negative control, representing the condition where no treatment was administered, serving as a control group. PC = positive control, representing the positive control group where a treatment of 5 μg/mL of chrysin was applied, serving as a reference for assessing the impact of the treatment with pure chrysin. (**C**) Representative images from the FACS experiments conducted after 48 h of treatment with Fe_3_O_4_@PEG500, inducing apoptosis in 61.7% of cells. (**D**) Representative images from the FACS experiments conducted after 48 h of treatment with Fe_3_O_4_@Chitosan500, inducing apoptosis in 36.8% of cells. Viable cells stain negative for both PI and Annexin V (Q4); early apoptotic cells stain positive for Annexin V and negative for PI (Q3); necrotic cells stain positive for PI only (Q1); and late apoptotic cells stain positive for both Annexin-V and PI (Q2). In order to make the text shorter we decided not include, because the image is self-descriptive.

Our study employed the MTT cell viability assay to assess the cytotoxic effects of Fe_3_O_4_ NPs coated with PEG and chitosan at a 1:1 nanoparticle (Fe_3_O_4_@PEG500 versus Fe_3_O_4_@Chitosan500) and drug ratio to explore their anti-cancer activity. The HCT-116 cell lines were treated with concentrations ranging from 1 mg to 25 µg/mL for 24 h with both types of NPs. The inhibitory concentration (IC50) for HCT-116 cells following a 24-h treatment was determined to be 16.68 µg/mL. For subsequent investigations, an optimal concentration of 5 µg/mL was chosen for both the PEG and chitosan-coated Fe_3_O_4_ NPs, which was below the IC50 value. The IC50 value indicated that extended treatments for 48 and 72 h exhibited significant toxicity to the cells, leading us to exclusively focus on a 24-h treatment duration. The cell viability studies demonstrated that PEG-coated Fe_3_O_4_ NPs (Fe_3_O_4_@PEG500) exhibited a significantly higher inhibition of cells compared to their chitosan-coated counterparts at the same concentration (P_NP_PEG_Chry_ vs. _NP_Chit_Chry_ = 0.0033) (Figure 5A).

Interestingly, the cell viability experiments revealed that PEG-coated Fe_3_O_4_ NPs without chrysin showed a significantly higher inhibitory effect than chitosan-coated Fe_3_O_4_ without chrysin at the same concentration (P_NP_PEG_ vs. _NP_Chit_ = 0.0471). Furthermore, both NPs coated with PEG and chitosan exhibited a suppressive impact on cell viability in the absence of chrysin (P_NP_PEG_ vs. _Control_ < 0.0001 and P_NP_Chit_ vs. _Control_ < 0.0001). The apoptosis assay revealed that following a 48-h treatment at 5 µg/mL, PEG-coated Fe_3_O_4_ NPs containing chrysin induced a significantly higher rate of apoptosis compared to chitosan-coated NPs (P_NP_PEG_Chry_ vs. _NP_Chit_Chry_ = 0.0004), whereas 24 h of treatment showed no significant difference. Specifically, the apoptotic response was more pronounced for the 1:1 PEG-chrysin ratio of 5 µg/mL Fe_3_O_4_@PEG500 than the 1:1 chitosan-chrysin ratio of 5 µg/mL Fe_3_O_4_@Chitosan500. These observations indicate that PEG-coated NPs have a more pronounced inhibitory effect on HCT-116 cells, underscoring their potential as a more effective cytotoxic agent in this context (Figure 5B). These findings contribute valuable insights into the differential cytotoxicity of coated Fe_3_O_4_ NPs, providing a basis for further exploration and optimization of nanoparticle formulations for enhanced anti-cancer applications.

## 4. Discussion

Optimizing nanoparticle coating conditions is essential for developing effective drug delivery systems, as interactions with biological fluids (blood, interstitial fluid, or mucosal secretions) lead to the formation of a protein layer, also known as a protein corona, around the nanoparticles, which can significantly influence their properties [41,42]. The development of strategies to produce nanoparticles with improved biocompatibility and lower immunogenicity is one of the main tasks of current studies, in which surface modifications to minimize protein adsorption and improve overall performance are one of the main directions [43].

Improving the loading efficiency of NPs by modifying their surface properties is a crucial aspect of enhancing the effectiveness of drug delivery systems [44]. Formulation parameters, including coating concentration, drug/carrier ratio, and nanoparticle synthesis method, need to be systematically optimized. Small changes to these parameters can have a significant impact on loading efficiency [45].

The modification of NPs with different coating agents shields the surface from aggregation [46]. Additionally, it alters their surface chemistry [47], reduces opsonization by proteins [48], and decreases phagocytosis by macrophages [49], thereby prolonging their circulation time. Moreover, particle surface modification can significantly influence their cellular uptake and toxicity.

In this study, we used a modified co-precipitation technique for the preparation of magnetic Fe_3_O_4_ nanoparticles containing PEG and chitosan as stabilizing polymers and loaded them with the anti-tumor agent chrysin. There are limited studies on the development and characterization of magnetic drug transport systems loaded with chrysin. Nosrati et al. (2018) reported the synthesis of chrysin-loaded iron oxide magnetic nanoparticles coated with L-phenylalanine (chrysin@Phe@IOMNs), demonstrating the potential application of co-polymers with more complex structures in coatings or more than one drug substance as an effective drug carrier [50]. Another study investigated the loading of doxorubicin and chrysin onto magnetite nanoparticles coated with a PCL–PEG–PCL triblock co-polymer [51]. This study explored the factors influencing drug-loading efficiency, revealing positive effects on solubility properties, gradual drug release, and enhanced anti-tumor effects. Notably, the literature lacks studies on chrysin loading and the system characterization of magnetite nanoparticles coated with PEG or chitosan. Therefore, we studied the structure and anti-tumor efficacy of the system by varying the concentration of drug and coating materials to determine their optimal effective proportion that maximizes drug loading while maintaining system stability. In our study, the most efficient loading was observed in the presence of PEG as the coating agent, while a lower percentage of drug loading was noted when the chitosan polymer was used. This discrepancy can be attributed to chitosan’s hydrophobic property [52] creating a non-homogeneous environment in water. The drug was loaded into magnetite NPs using a non-covalent method during sample preparation. It is known that non-covalent interactions are stronger in homogeneous and dispersed environments than in non-homogeneous environments [53].

The change in the size of the crystallites determines the degree of order of the system [54,55]. From this perspective, it can be concluded that the variation in the loaded amount of chrysin does not affect the degree of crystallinity of PEG-coated NPs and, therefore, the regularity of the system. These results align with the UV–Vis results, indicating effective drug loading. Thus, for Fe_3_O_4_ NPs coated with PEG, a two-fold increase in drug amount does not practically affect the loading efficiency. However, there is a 33 Å difference in the sizes of crystallites obtained at 1:0.5 (Sample: Fe_3_O_4_@Chitosan250) and 1:2 (Sample: Fe_3_O_4_@Chtiosan1000) concentrations of chitosan-coated Fe_3_O_4_ NPs and drug concentrations. Such a difference in crystallite size for different amounts of loaded drug substance led to a 13% difference in the drug-loading efficiency.

Our cell culture experiments utilizing HCT 116 colon cancer cells validated the superiority of PEG coating over chitosan. Interestingly, both PEG and chitosan-coated NPs exhibited a statistically significant anti-cancer effect, even in the absence of the anti-cancer drug chrysin, when compared to the negative control. The selective cytotoxicity of iron oxide NPs in cancer cells compared to normal cells is a known phenomenon, underscoring the importance of conducting further investigations to achieve a comprehensive understanding of the underlying mechanisms [56]. The mechanisms being studied extensively in this context include the enhanced permeability and retention (EPR) effect and the stimulation of magnetic hyperthermia in tumor tissues [57]. In direct comparison, PEG-coated Fe_3_O_4_ NPs demonstrated a stronger anti-cancer effect than their chitosan-coated counterparts. These findings underscore the potential of PEG-coated NPs as a more efficacious option for anti-cancer applications, warranting further exploration and consideration in the development of targeted cancer therapies.

PEG is a platform that provides several advantageous properties [58] within drug delivery, and one of them is ensuring the stability of nanosuspensions over a wide pH range. PEG-based nanostructures were also evaluated for extended circulation times [59]. The polysaccharides most widely used for modifying the surface of magnetic NPs include alginate, pullulan, chitosan, dextran, heparin, and starch [60,61]. Studies have reported that, for instance, the chitosan coating of Fe_3_O_4_ NPs leads to the formation of positively charged particles and enhances cellular uptake [62].

In conclusion, our study investigated the impact of coating agents on the structural properties and drug-loading efficiency of Fe_3_O_4_ NPs, specifically focusing on chitosan and PEG coatings. The XRD results revealed a notable trend: regardless of the type of coating agent, the presence of chrysin in varying amounts influenced the crystallite sizes. Remarkably, PEG-coated NPs consistently exhibited the smallest crystallite sizes even at the highest drug loading values, suggesting that the addition of chrysin did not disrupt the structural order of the PEG-coated system. This observation aligns with the loading efficiency results, indicating that PEG-coated NPs maintained their structural integrity and loading efficiency even with a two-fold excess of drug. In contrast, chitosan-coated NPs exhibited an increase in crystallite size with a corresponding rise in drug loading. This trend suggests that, unlike PEG, the regularity of the chitosan-coated system was affected by higher drug amounts, potentially impacting loading efficiency. Therefore, the optimal nanoparticle-to-drug ratio, ensuring maximum drug loading while maintaining structural regularity, was determined to be 1:1 for PEG-coated NPs. This finding highlights the robustness of PEG in preserving the order of the NP drug system even under increased drug-loading conditions. On the other hand, chitosan-coated NPs demonstrated a relative increase in loading efficiency with higher drug amounts, albeit at the expense of structural regularity. The potential consequence of reduced structural regularity in chitosan-coated systems was postulated to be an increased likelihood of recognition and removal by living organism cells as “dangerous” particles. This insight underscores the importance of considering not only the loading efficiency but also the structural impact of drug loading on the coated NPs, particularly for applications where the preservation of the nanoparticle structure is critical for therapeutic efficacy and safety. The beneficial role of PEG as a coating agent was emphasized in our cell culture experiments with HCT 116 colon carcinoma cells, wherein PEG-coated NPs exhibited a stronger inhibition of cell viability and a higher apoptosis rate compared to their chitosan-coated counterparts.

In summary, the selection of coating material plays a pivotal role in governing the structural response of drug-loaded NPs, with PEG demonstrating superior structural robustness even under high drug-loading conditions. These findings contribute valuable insights into the design and optimization of nanoparticle-based drug delivery systems for enhanced efficacy and biocompatibility.

## Figures and Tables

**Table 1:** The composition of Fe_3_O_4_ NPs. While preparing the samples, the quantity of Fe_3_O_4_ NPs coated with PEG and chitosan remained constant at 500 mg, while the amount of chrysin loaded into the NPs was varied at three different ratios: 1:0.5, 1:1, and 1:2.

Sample	Fe_3_O_4_ NPs (mg)	Coating Agent	Chrysin (mg)	Ratio of Fe_3_O_4_ and Chrysin
Fe_3_O_4_@PEG250	500	PEG	250	1:0.5
Fe_3_O_4_@PEG500	500	PEG	500	1:1
Fe_3_O_4_@PEG1000	500	PEG	1000	1:2
Fe_3_O_4_@Chitosan250	500	Chitosan	250	1:0.5
Fe_3_O_4_@Chitosan500	500	Chitosan	500	1:1
Fe_3_O_4_@Chitosan1000	500	Chitosan	1000	1:2

**Table 2:** The 2-theta angle values and crystallite sizes of iron oxide NPs coated with surface PEG and chitosan. The average crystallite sizes of magnetite NPs with different coatings were calculated according to Scherrer’s equation.

Samples	2-Theta Angle Values					Crystallite Size
Fe_3_O_4_@PEG	30.30°	35.66°	43.29°	57.17°	62.81°	25.30 nm
Fe_3_O_4_@Chitosan	30.37°	35.74°	43.45°	57.5°	62.83°	25.10 nm

**Table 3:** Results obtained from the UV–Vis and XRD analyses.

Sample	Crystallite Size (XRD)	Loading Efficiency (UV–Vis)
Fe_3_O_4_@PEG250	164 Å	92%
Fe_3_O_4_@PEG500	168 Å	96%
Fe_3_O_4_@PEG1000	153 Å	97%
Fe_3_O_4_@Chtiosan250	208 Å	45%
Fe_3_O_4_@Chtiosan500	192 Å	50%
Fe_3_O_4_@Chtiosan1000	175 Å	58%

## Data Availability

The data are contained within the article.
